# Acute Disease

**Published:** 1855-08

**Authors:** 


ACUTE DISEASE. *On the Diagnosis of Typhus and Typhoid Fever.*—By (1) Dr. PARKES, Physician to University College Hospital; (2) Prof. FORGET, of Strasburg; and (3) Dr. RITCHIE, Physician to the Royal Infirmary at Glasgow.

1. (*Medical Times and Gazette*, Nov. 24, 1844.)
2. (*Gaz. Méd. de Paris*, Nov. 25, 1854.)
3. (*Glasgow Medical Journal*, Oct. 1854.)

It appears to be still necessary to insist occasionally upon the non-identity of typhoid and typhus fever; and we therefore transcribe the following remarks:

1. Dr. Parkes speaks as follows in a clinical lecture:

"You are brought to see this young woman, we will say for the first time: the specific rose-spots are gone; she is laboring to all intents and purposes under severe bronchitic and chest symptoms (a chemist, or practitioner with a druggist's shop, has prescribed and given cough mixtures, perhaps, without seeing her); you find her respiration 30 in a minute, cough incessant, with some expectoration; nervous symptoms also well marked; vertigo complained of, torpor, the eyes closed; she is delirous at night; she has also diarrhœa, pain over the abdomen, pulse quick, tongue furrowed and somewhat coated. Suppose, I say, you were called to such a patient, and moreover she is unable to give any account of the previous illness, how are you to make the diagnosis? There are only two ways—one the positive method, the other the method as it is called by 'exclusion.' The first is obvious enough, and will of course be more valuable to the practiced eye of the experienced physician, who seizes the nature of the case at the first glance by a sort of intuitive knowledge of what typhoid really is. Now the method of diagnosis by exclusion—the plan of logic-writers, *per viam exclusionis*, in this and other diseases, is one, though not without disadvantages, yet of no mean importance. The first question you resolve in your mind will be—Is she or he, as the case may be, laboring under any of the idiopathic fevers? any of the exanthemata? No. Is it typhus? You make the same answer, as the eruption in ty-*phus* is as different from ty-*phoid* as scarlatina from measles. The eruption is absent in patients under 22 or 21 (this patient's age is about this). Is it relapsing fever, so common some years, as 1828–29? No. You ask yourself, then, is it typhoid? Yes. Nervous symptoms are marked, chest symptoms and diarrhœa also; the latter loose, granular, yellow, so peculiar to typhoid. You have soreness of the right iliac fossa; but then you say we have no rose-spots, and then you remember in at least 20 per cent. these rose-spots are not found. You must weigh and balance all these circumstances in your mind."

2. Dr. Forget has written several elaborate articles in the 'Gazette Médicale de Paris,' to furnish clinical proof of the non-identity of typhus and typhoid fevers.

In these articles he does not deny that typhoid fever may arise very commonly from infection; but he believes, from an experience of twenty years, that it may also proceed from a variety of causes quite foreign to it—such as moral affections, irregularities of diet, different morbid affections of both solids and liquids. Thus, follicular inflammation may come on from local causes, idiopathic, attacking primarily the digestive canal. Typhus never appears unless under some unfavorable conditions of the atmosphere, and then it first attacks a district occupied by persons closely aggregated, and in want of proper nourishment.

By the external general aspect, severe typhoid and typhus fevers resemble one another; but upon close inspection, certain points of difference will be observed. In follicular enteritis, the typhoid state is not constant, and is usually secondary. In typhus the stupor and the prostration exist from the invasion of the attack, indicating a general cause acting primarily as a powerful poison.

In typhoid fever, the gastro intestinal symptoms are more constant and more primitive, for they exist sometimes alone; and it is excessively rare to meet with cases of follicular enteritis which do not offer from the commencement the sandy, dotted, and rosy appearance of the tongue; the rumbling (*gargouillement*) and pain in the right iliac fossa, the diarrhœa or the constipation, etc. Other symptoms may dominate over these, but they do not absorb them completely; while in typhus, especially at the commencement, the tongue is very often humid, smooth, and white; the abdomen is exempt from tympanitis and pain, and defection is not sensibly altered. It is, nevertheless, true, that, in many instances, gastro-intestinal symptoms perfectly simulate those of follicular enteritis. Both typhus and typhoid fevers may be violently febrile or completely apyretic; either of them may present pectoral symptoms. As regards the lenticular rosy-spots, represented as proper to typhoid fever, it is proved that they may be produced in typhus. The author admits, however, that they constitute perhaps the best differential symptom. Typhoid fever is generally more slow and gradual in its progress. In simple, non-complicated cases, this uniform evolution is in exact relation to the development of the intestinal lesions. In typhus it is different; the disease acquires at the outset its greatest amount of intensity; then it oscillates, and varies from better to worse suddenly and unexpectedly. The nervous system has been acutely impressed by the morbid poison, and the ulterior accidents depend upon the variable localizations which happen to be produced. The duration of the diseases presents well-marked points of difference.

While typhoid fever passes through its stages gradually, and with a quasi-fatal regularity, typhus becomes suddenly milder or more severe, and, under most unfavorable-looking aspects, may run on to cure with remarkable rapidity.

As regards treatment, it need scarcely be said, that no specific remedy has been found for the arrest of typhoid fever; the treatment must depend upon the features of each particular case; bleedings and purgatives, chlorine and mercurials, musk and bark, have respectively their proper application. But the author attaches especial importance to the management of the ulcerated digestive tube, and he forbids the administration of such stimulants as would act violently, or of any strong medicine calculated to excite irritation. In typhus fever this precept does not apply, the intestinal lesion being absent or accidental. Whether the practioner has to deal with cerebral, pectoral, or abdominal typhus, he need not fear, by any plan of treatment he can adopt, that he will be aggravating the intestinal ulcerations.

The same careful and scientific practitioners who, bearing in mind the ulcerated or gangrenous state of Peyer's patches of glands in typhoid fever, cease to expect to found any system of treatment by which that disease may be cut short, do not despair of finding some agent sufficiently powerful to overcome the poison which in typhus fever is the cause of its characteristic train of symptoms. Nevertheless, we do not here possess any specific; but we may remove the patient from the infected district with advantage, and place him in pure air.

3. Dr. Ritchie's remarks are the conclusions to one of a set of clinical lectures on typhus and continued fever, which is published in the 'Glasgow Medical Journal.' He says:

"In conclusion, I would submit that, in the long course of inquiry now gone over, reaching from before Hippocrates to the present day, there is continuous evidence of the existence of two separate and essential, or primitive forms of fever, possessing many features of resemblance, and yet more of difference the one with the other, and which, ever and anon, gave occasion to discussion among medical men on their nature and relations. The one fever, or our typhus, being distinguished by independence of situation, season, or temperature, arising spontaneously from human affluvia, as in crowded camps or jails, but possessed of eminently contagious properties, and being often therefore imported from distant infected localities; having a measle like efflorescence on the skin as early as the fourth day, profound and diversified affections of the sensorium, great prostration of the vital powers, a disposition to putrescency, and requiring cordial food, wine, and other stimulants for its treatment. A crisis, in favorable cases, taking place on the fourteenth day, followed by a good recovery; but death often happening on the twelfth day, the necroscopical appearances being chiefly negative, or only such as are occasioned by fluidity of the blood, and by softening of the solids.

"The other fever, again, or our enteric, being of indigenous origin, arising in damp and cold seasons and countries as a simple sporadic fever, but, under special climatic and hygienic conditions, developing malignant epidemic, and also contagious qualities. Its prominent symptoms manifesting themselves much in the abdomen and thorax, the cutaneous eruption papular in form, inconstant, comparatively scanty, and appearing only about the eighth day; the disposition to crisis feeble, and seldom occurring before the twenty-first or twenty-eighth day, and the tendency to local complications so strong, that recovery often did not commence before the eightieth day. When death was the result, it was usually from inflammation, and sometimes perforation of the bowels, and the appearances on dissection were distinctive of inflammatory degeneration of the mucous membrane, the follicles, and other glands of the intestines. A moderately antiphlogistic, a soothing, cooling, and expectant treatment, such as one or two bleedings, a mild diet, fomentations to the belly, and abstinence from wine, spirits, or any kind of fermented liquor or stimulating food, was that which was suited to the disease."—*Half-Yearly Abstract of the Medical Sciences.*

